# Diagnosis and Surgical Treatment Outcomes of Cardiac Myxoma: Twenty Years of Data at a Single Institution

**DOI:** 10.3390/medicina61112025

**Published:** 2025-11-13

**Authors:** Gabriele Jakuskaite, Povilas Jakuska, Rimantas Benetis, Jolanta Justina Vaskelyte, Egle Ereminiene

**Affiliations:** 1 Department of Cardiology, Medical Academy, Lithuanian University of Health Sciences, LT-50161 Kaunas, Lithuania; 2 Kaunas Region Society of Cardiologists, Sukileliu pr. 15, LT-50103 Kaunas, Lithuania; 3 Institute of Cardiology, Lithuanian University of Health Sciences, LT-50161 Kaunas, Lithuania; 4 Department of Cardiac, Thoracic and Vascular Surgery, Medical Academy, Lithuanian University of Health Sciences, LT-50161 Kaunas, Lithuania

**Keywords:** primary cardiac tumour, cardiac myxoma, cardiac surgery

## Abstract

*Background and Objectives:* Cardiac myxoma (CM) is the most common primary benign neoplasm of the heart. This study’s objective was to analyse diagnostic features of CM, surgical data and postoperative courses of patients over a 20-year period in a single institution. *Materials and Methods:* We conducted a retrospective analysis of patients with diagnosed and pathologically confirmed CM who underwent surgical resection in our hospital from 1 January 2004 to 1 January 2024. Data was assessed and analysed from medical records. *Results:* The study included 76 patients (mean age, 61.7 ± 12.6 years; 60.5% female). The majority of patients (93.7%) had symptoms, most commonly presenting with dyspnoea (64.5%), chest pain (39.5%) and arrhythmias (35.5%). Myxomas were found in the left atrium (89.5%), right atrium (9.2%) and left ventricle (1.3%). Isolated tumour extirpation surgery was performed in 50 patients (65.8%). During the early postoperative period, arrhythmias were the most common complication (*n* = 16, 21.1%). Early in-hospital mortality occurred in two patients due to cardiopulmonary failure. In the late postoperative period, 11 deaths (14.9%) were observed 4 to 17.5 years after surgery. No recurrence of CM was found in any patient during the follow-up period, yet tumours of other localisations were detected in nine patients. *Conclusions:* Surgery is the first-line treatment for CM, with a good prognosis. Although during the late postoperative period no cardiac tumour recurrence was observed in our study, 12.2% patients were newly diagnosed with non-cardiac neoplasms. Therefore, we suggest monitoring patients not only for cardiac disorders but also for the occurrence of extracardiac tumours.

## 1. Introduction

Cardiac tumours are rare, especially primary neoplasms [1]. Up to 90% of primary cardiac neoplasms are benign, of which the most common is cardiac myxoma (CM), observed in 50% of cases [2,3]. The diagnosis of CM is based on imaging studies (echocardiography, cardiac computed tomography and/or magnetic resonance imaging) and is confirmed by histopathological evaluation [2,3]. Surgery remains as the first-line treatment for CM [3]. The aim of this study was to review 20 years of experience in diagnosing and surgically treating cardiac myxomas, as well as to assess long-term survival after surgical intervention.

## 2. Materials and Methods

A retrospective study of 76 adult patients (>18 years old) with diagnosed and pathologically confirmed cardiac myxoma who underwent surgical resection in our hospital over a 20-year period from 1 January 2004 to 1 January 2024 was conducted. Patients were included if they had a diagnosed cardiac myxoma, underwent surgical resection during the study period, and had histopathological confirmation of the myxoma diagnosis. Patients were excluded if the tumour was not resected, if histopathological examination was not performed, or if the results did not confirm cardiac myxoma. The primary endpoint of the study was overall survival following surgical resection of cardiac myxoma. Secondary endpoints included postoperative complications and tumour recurrence. Clinical signs and examination data, surgical treatment data and postoperative course were assessed and analysed from medical records. Follow-up data were retrieved from medical records and included documentation of postoperative visits, imaging results and survival status. The study received ethical approval from the Bioethics Center of Lithuanian University of Health Sciences (11 January 2021, no. BEC-MF-183). The need for patient consent was waived due to the use of retrospective data. All patient data were anonymised prior to analysis to ensure confidentiality.

### 2.1. Surgical Technique

All patients underwent median sternotomy and cardiopulmonary bypass with cannulation of both the inferior and superior vena cava. Then, aortic cross-clamping was performed and antegrade crystalloid (St. Thomas) cardioplegia was administered. In patients with atrial myxomas, right atriotomy was performed. Left-side tumours were reached via transatrial septum. However, the specific surgical approach was chosen depending on the localisation of the tumour and the need for other cardiac pathology treatment. Prior to undertaking the surgical management of other pathologies, the resection of the CM was performed as the primary procedure. To avoid fragmentation, gentle mobilisation of CM was performed before resections of the tumour. In cases of a significant atrial septal defect after tumour resection, closure was performed using an autologous pericardial patch.

### 2.2. Statistical Analysis

The data were analysed using MS Excel 2021 and SPSS 27.0 packages. Missing data were handled by complete case analysis; only available data were included in each analysis. Continuous variables were expressed as mean ± standard deviation (SD) or median (range), as appropriate. Categorical variables were presented as counts (*n*) and percentages (%). Differences between skewed variables were assessed using the Mann–Whitney U test. The Kaplan–Meier method was used to draw a survival curve. Results with *p* < 0.05 were considered statistically significant. Given the relatively small sample size (*n* = 76) and limited number of events, adjusted multivariable analyses were not performed to avoid model overfitting and unstable estimates. Therefore, the results should be interpreted as exploratory and descriptive.

## 3. Results

### 3.1. Clinical Characteristics

The study included 30 men (39.5%) and 46 women (60.5%). The mean age was 61.7 ± 12.6 years and did not differ significantly between gender groups. The frequency of comorbidities and risk factors are shown in Table 1. The most common comorbidities were arterial hypertension (*n* = 57, 75.0%) and dyslipidemia (*n* = 41, 54.0%). Notably, before the diagnosis of CM, 10 patients (13.2%) had been diagnosed with non-cardiac neoplastic diseases, of which 4 patients had uterine fibroids; the other 6 were diagnosed with intramuscular lipoma of the lower limb, liver hemangioma, lymphogranulomatosis, renal cell carcinoma, urinary bladder cancer and prostate adenocarcinoma, respectively.

### 3.2. Clinical Presentations

The majority of the study population were symptomatic, and only five patients (6.6%) were diagnosed with CM incidentally. No statistically significant difference in tumour size was found between symptomatic and asymptomatic patients (*p* = 0.342). Arrhythmias (*n* = 27, 35.5%), chest pain (*n* = 30, 39.5%) and dyspnoea (*n* = 49, 64.5%) were the most common symptoms presented by patients. However, less than a third (28.9%) of the study population were classified as III-IV functional class according to the New York Heart Association (NYHA). In total, symptoms caused by hemodynamic alterations were observed in 67 patients (88.2%). Non-specific symptoms appeared in 23 patients (30.3%), the most common of which was general weakness. Acute embolic symptoms were detected in six patients, of which five patients had embolisation in the blood vessels of the brain and one patient in the blood vessels of the limbs (leg). The frequency of symptoms is presented in Table 2.

### 3.3. Diagnostic Features

Transthoracic echocardiography (TTE) was performed in all patients. Moreover, additional imaging tests were performed in more than half of the patients (*n* = 41, 53.9%) to clarify the diagnosis: transoesophageal echocardiography (*n* = 30, 39.5%), cardiac magnetic resonance imaging (*n* = 12, 15.8%) and/or computed tomography (*n* = 8, 10.5%).

The vast majority of CM (*n* = 68, 89.5%) were found in the left atrium, with other locations including the right atrium (*n* = 7, 9.2%) and the left ventricle (*n* = 1, 1.3%). CM were mostly attached to the atrial septum (*n* = 70, 92.1%), with less common sites including the lateral inferior wall (*n* = 1) and the posterolateral wall (*n* = 1) of the left atrium, the free wall of the right atrium (*n* = 1) and the fundus (*n* = 1), the posterior leaflet of the mitral valve (*n* = 1) and the interventricular septum (*n* = 1). CM sizes varied widely from 8 mm to 80 mm in height and 7 mm to 55 mm in length. Hemodynamically significant mitral valve dysfunction was observed in six patients. Main echocardiographic characteristics are shown in Table 3.

All study patients underwent coronary artery angiography. Hemodynamically significant stenosis of coronary arteries was found in just over a fifth of patients (*n* = 16. 21.1%): eight patients had stenosis in one coronary artery, three patients were diagnosed with double vessel and five patients with triple vessel coronary artery disease.

### 3.4. Surgical Treatment and Postoperative Period

**Figure 1:**
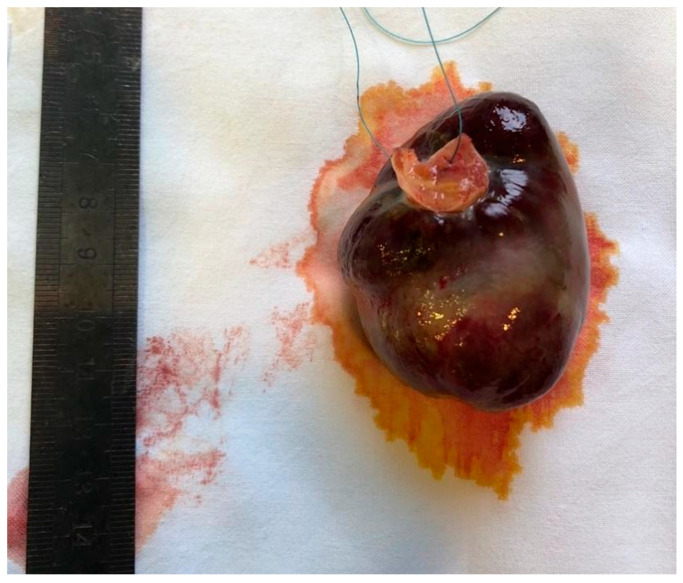
Cardiac myxoma after removal from the left atrium.

**Figure 2:**
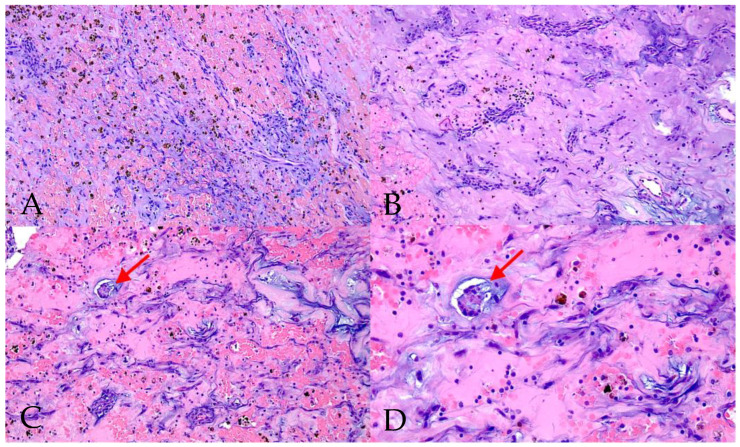
Microscopic histological image of cardiac myxoma (haematoxylin and eosin stain) showing typical myxoid cells with eosinophilic cytoplasm. Proliferation of cells, forming cords and nests, is marked with arrows ((**A**–**C**)—magnification ×10, (**D**)—magnification ×20).

All patients underwent surgical tumour extirpation, and histopathological confirmation of CM diagnosis was performed. Macroscopic and microscopic images of CM are presented in Figure 1 and Figure 2.

Isolated tumour resection was performed in almost two-thirds of patients (*n* = 50, 65.8%). Complex surgery was performed in 26 patients: in addition to tumour removal, 16 patients required coronary artery bypass grafting (CABG), and 12 patients required surgical valve correction (12 tricuspid valve repairs, three mitral valve repairs and one aortic valve replacement).

During the early postoperative period, 16 patients (21.1%) developed new arrhythmias or conduction disturbances. As a result, only two patients (2.6%) needed long-term medical therapy or pacemaker implantation, whereas arrhythmias were transient in the remaining 14 patients. Interestingly, of the 27 patients previously diagnosed with rhythm disorders, 12 patients had no arrhythmias following CM resection. Other early postoperative complications were seen in 14 patients (18.4%): pneumonia (*n* = 6, 7.9%), pleural effusion (*n* = 3, 3.9%), operative wound infection (*n* = 3, 3.9%), acute renal failure (*n* = 2, 2.6%) bleeding (*n* = 3, 3.9%) and postoperative anaemia (*n* = 4, 5.3%). In-hospital mortality was observed in two patients (2.6%) who died from cardiopulmonary insufficiency.

For the remaining 74 survivors, a long-term postoperative course was observed from the date of surgery to the date of death or 1 January 2024 if the patient was alive. The mean follow-up was 8.3 ± 5.9 years, ranging from 2 months to 20 years after surgery. During the follow-up period, no recurrence of CM was observed. However, nine patients (12.2%) were newly diagnosed with non-cardiac neoplasms, of which four were malignant tumours (diagnosed 2 to 17 years after CM surgery). Newly diagnosed neoplasms were non-small-cell lung cancer for two patients (adenocarcinoma and squamous-cell carcinoma), cervical cancer, facial basal-cell carcinoma, adrenal adenoma, uterine leiomyoma, low grade colon dysplasia, intraductal papillary mucinous neoplasm and liver hemangioma. The time of diagnosis of extracardiac neoplasms after surgery is presented in Table 4.

**Figure 3:**
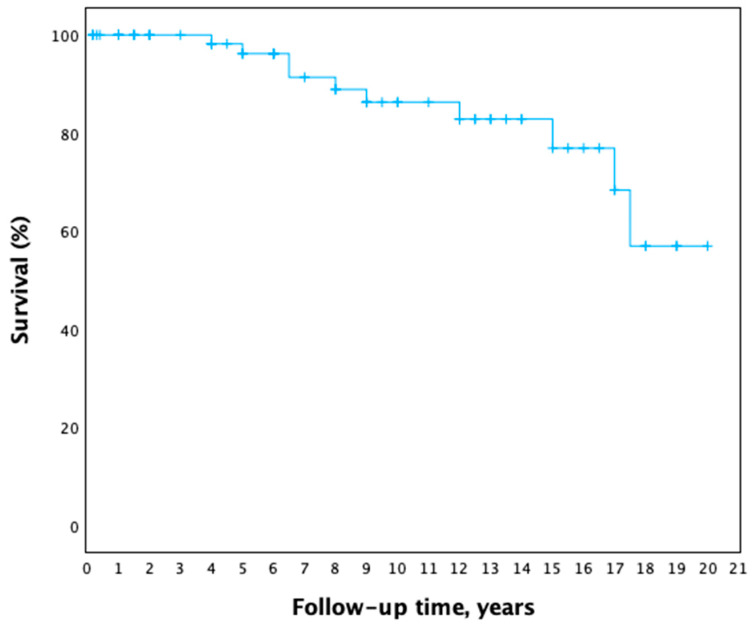
Late postoperative survival curve (Kaplan–Meier).

During follow-up, 11 patient deaths were registered, ranging from 4 years to 17.5 years after surgery (median 8.5 years). The age of the deceased patients ranged from 61 years to 85 years, median—72 years. Even though most of the patients’ cause of death was unknown, three patients died from non-cardiac oncological disease progression, two patients from metastatic lung cancer and one from metastatic cervical cancer. The late postoperative survival curve is shown in Figure 3. No differences in survival were found between age or sex groups.

## 4. Discussion

Cardiac tumours are rare, especially primary neoplasms, which account for 0.001–0.3% of cases in autopsy studies [1]. CM is the most common primary neoplasm of the heart [1,2]. In our study, we observed the predominance in female patients, and this distribution echoes the literature, which suggests that CM are 1.5–2 times more common in women than in men [4]. Our study population was slightly older (with a mean age of 61.7 ± 12.6 years) than indicated by various reports in the literature, which could be due to the smaller sample size [5,6].

The intracardiac localisation of myxomas reflects the general trend reported in the literature: the vast majority of these tumours are observed in the left atrium (89.5% in our study), less frequently in the right atrium and least commonly in the ventricles [5,7]. The most common site of attachment is the interatrial septum, usually at the fossa ovalis [5,7]. Up to 10% of CM may be associated with Carney complex or can be seen in familial cases; however, most of CM occur sporadically [2].

Although CM can be diagnosed incidentally, this cardiac neoplasm usually manifests through three major symptom groups: nonspecific signs (general weakness, fever, weight loss, arthralgia and myalgia), obstructive symptoms (dyspnoea, chest pain, arrhythmias, syncope, chronic heart failure symptoms) and/or embolic (cerebral or peripheral) events [1]. Since CM is a benign neoplasm, the severity and frequency of these symptoms largely depend on the location, mobility and/or size of the tumour [5]. In our study, obstructive symptoms were the most common, observed in 67 patients (88.2%), with dyspnoea being a dominant one (64.5%).

The initial evaluation of a cardiac tumour is performed using multimodality imaging [8]. Because of its wide availability, two-dimensional TTE is usually used as the first diagnostic approach [8]. If needed, it may be followed by transoesophageal echocardiography (TEE) because of its sensitivity and accuracy [8,9,10]. Advanced imaging techniques, such as computed tomography (CT), magnetic resonance imaging (MRI) and positron emissions tomography (PET), are increasingly used in clinical practice for their non-invasive evaluation of the tumour and its potential involvement with surrounding cardiac structures [3,8,10]. Although in our study only 20 patients underwent MRI and/or CT imaging, it has been observed that the frequency of performing these imaging tests has been increasing in recent years. Nonetheless, histopathological examination of CM remains the gold standard for confirming the diagnosis [3,8].

Surgery is the first-line treatment for CM and should be performed without delay after diagnosis due to the high risk of embolisation and sudden death [3,5,7]. Moreover, all patients should undergo a detailed preoperative examination including coronary artery angiography, which provides information not only about CA stenosis but also about tumour blood supply and involvement with the CA [1,9]. In our study, all patients underwent a comprehensive preoperative examination; therefore, 26 patients required additional procedures (CABG, valve correction) during surgery besides tumour extirpation. The extent of surgery and the frequency of additional procedures vary between studies due to differences in age and comorbidities of the study populations [5,6].

Arrhythmia after cardiac surgery is a known and relatively common complication [11]. However, only two patients in our study required long-term medical treatment or pacemaker implantation for newly developed arrhythmias and/or conduction disturbances. The literature states that early (30-day) mortality rates following CM resection ranges between 0% to 10% in previous studies [9]. Similarly, we observed in-hospital mortality in two patients (2.6%). In our study, no recurrence of CM was observed during the follow-up period, whereas various authors report different CM recurrence rates ranging up to 5.6% for sporadic cases [5,6,12].

The literature focuses on the importance of genetic testing to elucidate familial and related cases of Carney syndrome, thereby predicting the course of the disease and planning appropriate follow-up [5,6,13]. It is believed that in the case of genetic alterations responsible for the development of CM, benign and/or malignant tumours of other localisation may develop more often; thus, genetic testing would be extremely relevant in these patients [12].

In contrast to prior work on primary benign cardiac tumours, our findings highlight a noteworthy emergence of extracardiac neoplasms in the late postoperative phase. Although the studies reviewed did not describe the occurrence of non-cardiac tumours, we want to emphasise that in our study, nine patients (12.2%) were newly diagnosed with non-cardiac neoplasms, of which four were malignant tumours. This observation invites a re-evaluation of conventional monitoring protocols and suggests the need for extended surveillance strategies beyond the cardiac focus. Consequently, we suggest that during the follow-up period patients should be actively monitored not only for CM recurrence but possible non-cardiac neoplasm development. This long-term analysis prompts a broader discussion on oncological vigilance in cardiac tumour survivors—underscoring the interplay between cardiac and extracardiac pathology over time.

## 5. Conclusions

CM is a benign neoplasm, which manifests through three major symptom groups: nonspecific signs, obstructive symptoms and/or embolic events. The severity and frequency of these symptoms largely depend on the location, mobility and/or size of the tumour. TTE is the first-line diagnostic approach; however, additional imaging such as TEE, cardiac CT, MRI and PET are increasingly used in clinical practice. Surgery is the first-line treatment for CM with a good prognosis. Although no cardiac recurrences were observed during the late postoperative period in our study, 12.2% patients were newly diagnosed with non-cardiac neoplasms. Therefore, we recommend monitoring patients not only for cardiac disorders but also for the occurrence of extracardiac tumours.

### Limitations

We believe this study has its limitations. First, it was a retrospective study. Second, since cardiac tumours are rare, the study sample is not large. Third, there was no strict schedule during the follow-up period. Finally, although several non-cardiac neoplasms were observed during this study, genetic testing was not performed in any of the cases. Additional research including genetic testing is required.

## Figures and Tables

**Table 1:** Comorbidities and risk factors of the study population.

Comorbidities and Risk Factors	All Patients (*n* = 76) N (%)
Arterial hypertension	57 (75.0)
History of myocardial infarction	5 (6.6)
Cerebral stroke	4 (5.3)
Diabetes mellitus	6 (7.9)
Non-cardiac neoplastic diseases	10 (13.2)
COPD	8 (10.5)
Dyslipidaemia	41 (53.9)
Obesity	28 (36.8)

COPD—chronic obstructive pulmonary disease.

**Table 2:** The frequency of symptoms.

Symptoms		All Patients (*n* = 76) N (%)
Generalized/nonspecific symptoms:		23 (30.3)
	Fever	4 (5.3)
	Weight loss	5 (6.6)
	General weakness	17 (22.4)
	Arthralgia, myalgia	2 (2.6)
Obstructive cardiac symptoms:		67 (88.2)
	Dyspnoea	49 (64.5)
	Functional classification according to the NYHA:	
	Class I	11 (14.5)
	Class II	43 (56.6)
	Class III	15 (19.7)
	Class IV	7 (9.2)
	Chest pain	30 (39.5)
	Arrythmias	27 (35.5)
	Syncope	7 (9.2)
	Chronic heart failure symptoms	5 (6.6)
Peripheral embolisms:		6 (7.9)
	Cerebral stroke	5 (6.6)
	Peripheral (limb) emboli	1 (1.6)

NYHA—New York Heart Association.

**Table 3:** Main echocardiographic characteristics.

Echocardiographic Characteristics	All Patients (*n* = 76)
Myxoma location	
Left atrium, *n* (%)	68 (89.5%)
Right atrium, *n* (%)	7 (9.2%)
Left ventricle, *n* (%)	1 (1.3%)
Size of myxoma	
Dimension 1 (height), mm ± SD (range)	37.8 ± 16.1 (8–80)
Dimension 2 (length), mm ± SD (range)	26.4 ± 11.4 (7–55)
LV ejection fraction (EF):	
≥50%, *n* (%)	67 (88.2)
30–49%, *n* (%)	7 (9.2)
<30%, *n* (%)	2 (2.6)
Mitral stenosis	62 (81.6)
Mild (Gmean < 5 mmHg), *n* (%)	52 (68.4)
Moderate (Gmean 5–10 mmHg), *n* (%)	7 (9.2)
Severe (Gmean > 10 mmHg), *n* (%)	3 (4.0)
Mitral regurgitation	63 (82.9)
Mild-moderate (I–II degree), *n* (%)	60 (78.9)
Moderate-severe (III–IV degree), *n* (%)	3 (4.0)
Tricuspid regurgitation	60 (78.9)
Mild-moderate (I–II degree), *n* (%)	53 (69.7)
Moderate-severe (III–IV degree), *n* (%)	7 (9.2)
Pulmonary hypertension (PASP > 40 mmHg), *n* (%)	15 (19.7)
LVEDD, mm ± SD	48.1 (±6.7)
LA size, mm ± SD	42.1 (±5.9)

EF—ejection fraction, Gmean—mean gradient, LA—left atrium, LV—left ventricle, LVEDD—left ventricle end-diastolic diameter, PASP—pulmonary artery systolic pressure.

**Table 4:** Timing of extracardiac neoplasm diagnosis following CM surgery.

Malignant Tumours	Time After CM Surgery	Benign Neoplasms	Time After CM Surgery
Cervical cancer	17 years	Intraductal papillary mucinous neoplasm	11 years
Lung cancer (squamous-cell carcinoma)	14 years	Low-grade colon dysplasia	10 years
Facial basal-cell carcinoma	5 years	Adrenal adenoma	1 year
Lung cancer (adenocarcinoma)	2 years	Uterine leiomyoma	1 year
		Liver haemangioma	1 year

## Data Availability

Derived data supporting the findings of the study are available upon reasonable request from the corresponding author.

## Abbreviations

The following abbreviations are used in this manuscript:

CABG	coronary artery bypass graft
CM	cardiac myxoma
CODP	chronic obstructive pulmonary disease
CT	computed tomography
EF	ejection fraction
Gmean	mean gradient
LA	left atrium
LV	left ventricle
LVEDD	left ventricle end-diastolic diameter
MRI	magnetic resonance imaging
NYHA	New York Heart Association
PASP	pulmonary artery systolic pressure
PET	positron emissions tomography
SD	standard deviation
TEE	transoesophageal echocardiography
TTE	transthoracic echocardiography
